# Isolation, Identification, Biological Characterization, and Pathogenicity of Entomopathogenic Fungus from the Larvae of the *Evergestis extimalis* (Scopoli) (Lepidoptera: Pyralidae)

**DOI:** 10.3390/biology14050467

**Published:** 2025-04-25

**Authors:** Youhua Ma, Minggang Qin, Yuanfang Zeng, Yinyin Shen, Youpeng Lai, Guangxin Lu

**Affiliations:** 1 Key Laboratory of Agricultural Integrated Pest Management of Qinghai Province, Qinghai Academy of Agricultural and Forestry Sciences, Qinghai University, Xining 810016, China; m2218937@163.com (Y.M.); 18189603228@163.com (M.Q.); 18787710121@163.com (Y.Z.); syy5647382910@163.com (Y.S.); 2 College of Agriculture and Animal Husbandry, Qinghai University, Xining 810016, China

**Keywords:** *Evergestis extimalis*, entomopathogenic fungi, *Mucor hiemalis*, cultivation conditions, pathogenicity

## Abstract

At Qinghai agriculture districts, *Evergestis extimalis* poses a significant threat to spring rapeseed cultivation through its larvae burring into the rapeseed kernels and feeding seeds. To protect the ecological environment of the Qinghai–Tibet Plateau, it is essential to research and develop biological control technologies for pest management. In this study, we isolated and purified a new entomopathogenic fungus from the carcasses of *E. extimalis* larvae, which was identified as *Mucor hiemalis* based on morphological characteristics combined with ITS rDNA and 18S rDNA sequence analyses. Subsequently, the optimal growth conditions for the strain were determined as follows: SDAY medium, fructose as the carbon source, peptone as the nitrogen source, 25 °C, pH 6.0–7.0, and a 0:24 (light:dark) photoperiod.However, UV can significantly reduce fungal spore production. The bioassay result shows its pathogenicity was a concentration-dependent effect on *E. extimalis*, and younger larvae were more susceptible. With 1 × 10^8^ spores/mL inoculated, survival of second instar larvae decreased by the greenhouse pot experiment. In conclusion, *M. hiemalis* exhibits a significant biocontrol potential against *E. extimalis*.

## Simple Summary

*Evergestis extimalis* is a significant pest of rapeseed crops. During our study on the biology of *E. extimalis*, we discovered a strain of pathogenic fungus that infected its larvae. Through morphological and molecular biological analyses, the strain was identified as *Mucor hiemalis* and named *M. hiemalis* QH01. Afterwards, the optimal growth conditions for strain QH01 were determined by evaluating mycelial growth rate and sporulation yield as indicators. We evaluated the pathogenicity of *M. hiemalis* against the larvae of the *E. extimalis* under laboratory conditions. The results indicated that the strain was highly pathogenic to the younger larvae, followed by the older larvae. Subsequently, the pathogenicity of the strain against the second instar larvae of the *E. extimalis* was further evaluated under greenhouse conditions. The results showed that the survival rate of the second instar larvae decreased significantly. Our findings will contribute to the development of *M*. *hiemalis* for the biological control of *E. extimalis*.

## 1. Introduction

*Evergestis extimalis* Scopoli (Lepidoptera: Pyralidae), a destructive pest, is mainly distributed in China, Korea, Japan, and the United States. It primarily infests rapeseed, fennel, cabbage, kale, and sugar beet crops. Its larvae burring into the rapeseed kernels and eating seeds cause serious harm to spring rapeseed, while the insect harms other host plants by leaf-rolling and eating core leaves and seed buds [1,2]. The spring rapeseed crop has the largest planting area in Qinghai Province, China, which was the primary income resource for local famers. However, the occurrence of *E. extimalis* has seriously threatened rapeseed production in Qinghai, causing great economic losses in recent years [1]. At present, *E. extimalis* is mainly controlled by chemical pesticides, which is limited by the emergence of strong resistance, environmental pollution, and concerns for human and animal health. Therefore, it is urgent to explore new alternatives for the management of *E. extimalis*, including a novel high-efficiency, low-toxicity, and low-residue microbial insecticide.

Entomopathogenic fungi (EPF) widely exist in nature and can infest insects, causing disease or even death [3]. They are considered highly effective in regulating the population of many Lepidoptera insect pests [4,5,6]. Shehzad et al. [7] reported strong pathogenicity of *Beauveria bassiana* and *Metarhizium anisopliae* against the second and third instar of *Plutella xylostella* under both direct spraying of inoculum and leaf dipping treatments. *M. anisoplariae* CQMa128 was shown to have a good control effect against the fourth instar larvae of *Pyrausta nubilalis* and *Pieris rapae*, with up to 95% and 76.7% mortality rates, respectively [8]. EPF of *E. extimalis* has rarely been reported in the literature. EPF may rapidly germinate and grow as a biological control agent [9]. However, the germination, growth, pathogenicity, and persistence of EPF can vary in different hosts and may be influenced by the strain types and environmental conditions [10]. For example, the growth and sporulation of most EPF usually reach their maximum at about 25 °C [11,12,13], and the maximum virulence is generally achieved at 20–25 °C [14].

*Mucor* spp. are widely distributed in nature, parasitizing plants and animals or exhibiting saprophytism in soil, plant, and animal residues, as well as in other substrates [15,16]. Some mucor species can infect certain Lepidoptera, Coleoptera, and Diptera insects [17]. Heitor et al. [18] isolated a virulent strain of *M. hiemalis* from *Mamestra brassicae* larvae. Reiss et al. [19] found *M. hiemalis* in *Artemia salina*. Konstantopoulou and Mazomenos [20] isolated four EPF from diseased pupae of *Bactrocera oleae* and larvae of *Sesamia nonagrioides*, and two strains of *M. hiemalis* were reported to have a good controlling effect on the adult of *Ceratitis capitata*. Konstantopoulou et al. [21] reported that the metabolic extract of *M. hiemalis* SMU-21 has insecticidal activity.

In this study, we identified a fungus infecting the larvae of *E. extimalis* and evaluated its pathogenicity. Our results show that it could be a potential biological control agent for the control of *E. extimalis*. Meanwhile, to protect the ecological environment of the Qinghai–Tibet Plateau, it is essential to research and develop biological control technologies for pest management.

## 2. Materials and Methods

### 2.1. Isolation of Entomopathogenic Fungus

The EPF strain was isolated from the carcasses of *E. extimalis* larvae, which were collected from Datong County (36°43′ N/100°51′ E), Qinghai Province, China. Briefly, the carcasses were soaked in 0.3% sodium hypochlorite for 1 min, washed with distilled water thrice, and then placed in a disposable Petri dish lined with sterile filter paper for moisturizing the culture [22]. When mycelia or spores were grown, a few were isolated in a sterile environment to inoculate on PDA (Potato Dextrose Agar) medium and cultured in an incubator at 23 ± 1 °C in the dark for 3 days. The next generation of mycelium was transferred into a new PDA plate and cultured five times to obtain the final isolate.

### 2.2. Morphological Characterization and Molecular Identification of the Isolated Fungus

The fungal isolate was inoculated on PDA medium, with a sterile and obliquely inserted coverslip, at 25 °C for 3 days. Subsequently, the grown mycelium was stained with lactic acid phenol cotton blue staining solution for morphological observation under an optical microscope. The pathogen was imaged and morphologically identified according to the Fungal Identification Manual [23].

A fungal genomic DNA extraction kit (Sangon Biotech, Shanghai, China) was used to extract the fungal DNA. Universal primers ITS1 (5′-TCCGTAGGTGAACCTGCGG-3′) and ITS4 (5′-TCCTCCGCTTATTGATATGC-3′) were used to amplify the ribosomal DNA internal transcriptional spacer (ITS) of the isolate. The small subunit 18S ribosomal DNA was amplified with NS1 (5′-GTAGTCATATGCTTGTCTC-3′) and NS6 (5′-GCATCACAGACCTGTTATTGCCTC-3′) primers. The primers were obtained from Shanghai Sangon Biological Co., Ltd., Shanghai, China. The PCR amplification reaction system consisted of 1 μL template DNA, 1 μL primers, 12.5 μL PCR MasterMix, and 9.5 μL dd H_2_O. The reaction conditions were as follows: pre-denaturation at 95 °C for 5 min, 30 cycles of denaturation at 94 °C for 30 s, annealing at 57 °C for 30 s, extension at 72 °C for 90 s, and a final extension at 72 °C for 10 min. The amplified products were detected and recovered by gel electrophoresis and sent to Shanghai Sangon Biological Co., Ltd., Shanghai, China, for sequencing. The sequencing results of the isolate were submitted to the GenBank nucleic acid sequence database for BLAST (https://ftp.ncbi.nlm.nih.gov/blast/) comparison, and sequences with higher homology were selected to construct a Neighbor-joining (NJ) phylogenetic tree with 1000 bootstrap calculations using MEGA 7.0 [24].

### 2.3. Optimization of the Culture Conditions of the Isolated Fungus

#### 2.3.1. Influence of Medium on Fungal Growth and Sporulation

Fungal growth rate and spore production were evaluated in various media (*n* = 8) as described in Table S1. The 8 mm diameter agar plug containing mycelia was inoculated in the plate center of the different test media, and the culture was performed in the dark at 25 °C for 3 days. The colony diameter was measured by the cross-crossing method every day to estimate the fungal growth rate. Each test was repeated three times. After 6 d of culture, the spores were eluted in 10 mL of sterile water, and the solution was filtered through four layers of gauze to make a spore suspension. The spores were estimated by counting using a blood cell counting plate under an optical microscope.

#### 2.3.2. Optimizing the Carbon and Nitrogen Sources for Fungal Growth and Sporulation

Using PDA as the base medium, glucose was replaced with fructose, sucrose, inositol, and maltose in equal amounts. The control group had no carbon source. Organic peptone was used as a nitrogen source, which was then replaced with equal amounts of sodium nitrate, ammonium sulfate, ammonium nitrate, and potassium nitrate. The control group contained no nitrogen source. Agar plugs containing mycelia (d = 8 mm) were cultured on media with different carbon and nitrogen sources at 25 °C in the dark for 3 days. All tests were repeated three times. The fungal growth rate and sporulation were calculated as described in Section 2.3.1.

#### 2.3.3. Optimization of Temperature for Fungal Growth and Sporulation

Agar plugs containing mycelia (d = 8 mm) were inoculated on optimized PDA plates at 15, 20, 25, 30, and 35 °C, as described in Section 2.3.1, and the fungal growth rate and sporulation were calculated. All tests had three repeats.

#### 2.3.4. Effect of pH on Fungal Growth and Sporulation

The pH values were adjusted to 5.0, 6.0, 7.0, 8.0, 9.0, and 10.0 with 1 mol/L HCl or 1 mol/L NaOH. Agar plugs containing mycelia (d = 8 mm) were inoculated on PDA media with different pH values, as described in Section 2.3.1. Average fungal growth rate and sporulation were calculated from three repeats at different pH values.

#### 2.3.5. Optimization of Photoperiod for Fungal Growth and Sporulation

The incubator was programmed with four light/dark (L:D) cycles: 24:0, 16:8, 12:12, and 0:24, while the rest of the fungal culture conditions were the same as in Section 2.3.1. Average fungal growth rate and sporulation were estimated from three repeats at different photoperiods.

#### 2.3.6. Effect of Ultraviolet (UV) Irradiation on Fungal Growth and Sporulation

The 8 mm diameter agar plugs containing mycelium were inoculated on PDA plates, which were placed at a vertical distance of 40 cm from a 30 W UV-C lamp in an ultra-clean bench. The lids of the Petri dishes were removed, and the plates were exposed to UV-C radiation for 5, 10, 15, 20, and 25 min. Each treatment was repeated 3 times. After the UV-C treatment, plates were cultured as described in Section 2.3.1, and the fungal growth and spore production rates were calculated.

### 2.4. Pathogenicity of EPF

#### 2.4.1. Rearing of Insects and EPF Treatment

The insect eggs and larvae were collected from Datong County, Xining City, Qinghai Province, China, and brought back to the laboratory in a self-made incubator (8 cm × 14 cm × 5 cm). The larvae were fed with fresh cabbage (*Brassica rapa*) in an artificial climate incubator (temperature 22 ± 1 °C, relative humidity 70 ± 5%). Fungal isolates were cultured on SDAY medium for 8 days at 25 °C, after which they were used to prepare 1 × 10^5^, 1 × 10^6^, 1 × 10^7^, and 1 × 10^8^ spores/mL aqueous spore solution (containing 0.05% Tween 80) under aseptic conditions. An aqueous solution containing 0.05% Tween 80 was used as a blank control.

#### 2.4.2. Bioassay Test

The dipping method was utilized for bioassay treated with the 1st to 5th instar larvae. The larvae (*n* = 15) were immersed in different spore suspensions for 20 s and then picked out to dry naturally. Subsequently, the larvae were transferred to an incubator (25 ± 1 °C, RH 70 ± 5%) and fed with fresh cabbage. Each treatment was repeated four times, and the mortality of larvae was observed every day.

#### 2.4.3. Greenhouse Pot Experiment

Several pots of rapeseed (Qingza No. 5) were planted in the greenhouse of the Qinghai Academy of Agriculture and Forestry, China. Finally, 16 healthy well-grown rapeseed pots were selected at the seedling stage. Healthy 2nd instar larvae of *E. extimalis* were carefully transferred to the plants using a soft brush, 15 larvae per pot. Spore suspensions were sprayed on the potted plants in low to high concentrations (1 × 10^5^, 1 × 10^6^, 1 × 10^7^, and 1 × 10^8^ spores/mL), ensuring coverage of larval cuticle. There were four replicates for each spore concentration, and the sterile aqueous solution containing 0.05% Tween 80 was used as a blank control. Survival rate was calculated on the third and fifth days of treatment.

### 2.5. Data Analysis

Equations (1)–(3) were used to calculate *E. extimalis* larval cumulative mortality, survival, and cumulative corrected mortality. SPSS 26.0 and GraphPad Prism 8.0 were used for statistical analysis. Analysis of variance (ANOVA) and least significant difference (LSD) tests were performed for fungal growth, spore production, and insect mortality rates. LC_50_, LT_50_, and their 95% confidence limits (CI) were calculated by probit probability analysis.

(1) $$\begin{array}{c} Cumulative\;mortality=\frac{\mathrm{Number}\;\mathrm{of}\;\mathrm{dead}\;\mathrm{insects}}{\mathrm{Total}\;\mathrm{number}\;\mathrm{of}\;\mathrm{treated}\;\mathrm{insects}}\times 100 \end{array}$$

(2) $$\begin{array}{c} Survival\;rate=\frac{\mathrm{Number}\;\mathrm{of}\;\mathrm{survival}\;\mathrm{insects}}{\mathrm{Total}\;\mathrm{number}\;\mathrm{of}\;\mathrm{treated}\;\mathrm{insects}}\times 100 \end{array}$$

(3) $$\begin{array}{c} Cumulative\;corrected\;mortality=\frac{\mathrm{Cumulative}\;\mathrm{mortality}\;\mathrm{of}\;\mathrm{treatment}\;\mathrm{group}\;-\;\mathrm{Cumulative}\;\mathrm{mortality}\;\mathrm{of}\;\mathrm{control}\;\mathrm{group}}{100\;-\;\mathrm{Cumulative}\;\mathrm{mortality}\;\mathrm{of}\;\mathrm{control}\;\mathrm{group}}\times 100 \end{array}$$

## 3. Results

### 3.1. Identification of the EPF Strain

**Figure 1:**
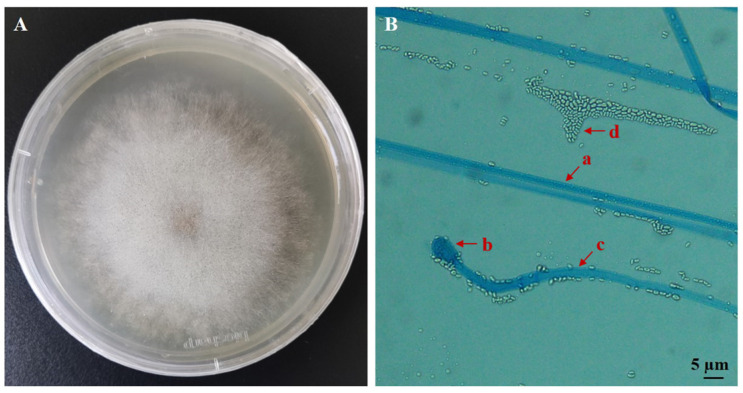
Morphological characteristics of the fungus on PDA medium. (**A**) Colony morphology after 3 days of growth on PDA at 25 °C. (**B**) Mycelia (a), sporangia (b), sporangiophore (c) and sporangiospores (d) under an optical microscope.

The fungus was cultured on a PDA medium for 3 days. The grown mycelium was white and feathery, with a dense center and sparse edges. Black sporangia could be observed at the top of the mycelium (Figure 1A). The fungus has 3.2–8.0 µm wide aseptate hyphae, and upon maturation of the sporangia releases ellipsoidal sporangiospores measuring 1.26–3.0 × 1.1–2.2 µm (Figure 1B).

**Figure 2:**
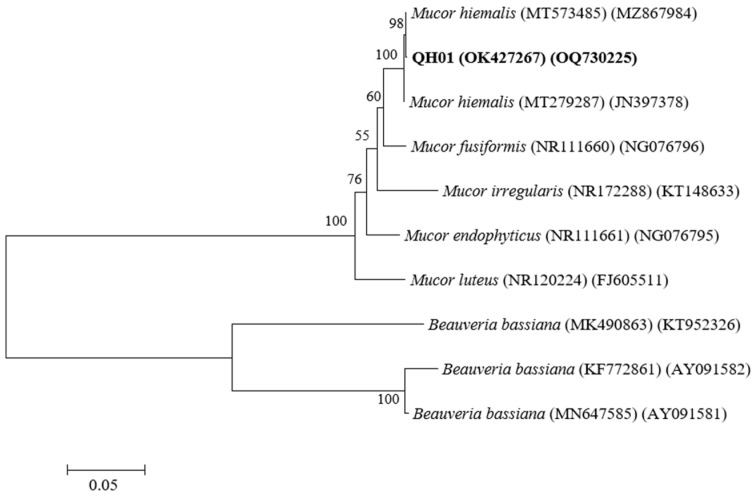
A NJ phylogenetic tree was constructed based on ITS and 18S gene sequences. Each isolate is marked with a GenBank accession number and the species name. Posterior probabilities are presented above the branches. The *M. hiemalis* isolated from *E. extimalis* in this study is marked in bold.

The 535 bp ITS sequence of the fungal isolate shares 100% homology with *M. hiemalis* (such as MT573485 and MT279287). Additionally, the 1353 bp 18S sequence shares more than 99% homology with *M. hiemalis*. We named the strain *M. hiemalis* QH01 (GenBank accession numbers OK427267 and OQ730225) (Figure 2).

### 3.2. Culture Condition Optimization of M. hiemalis

**Figure 3:**
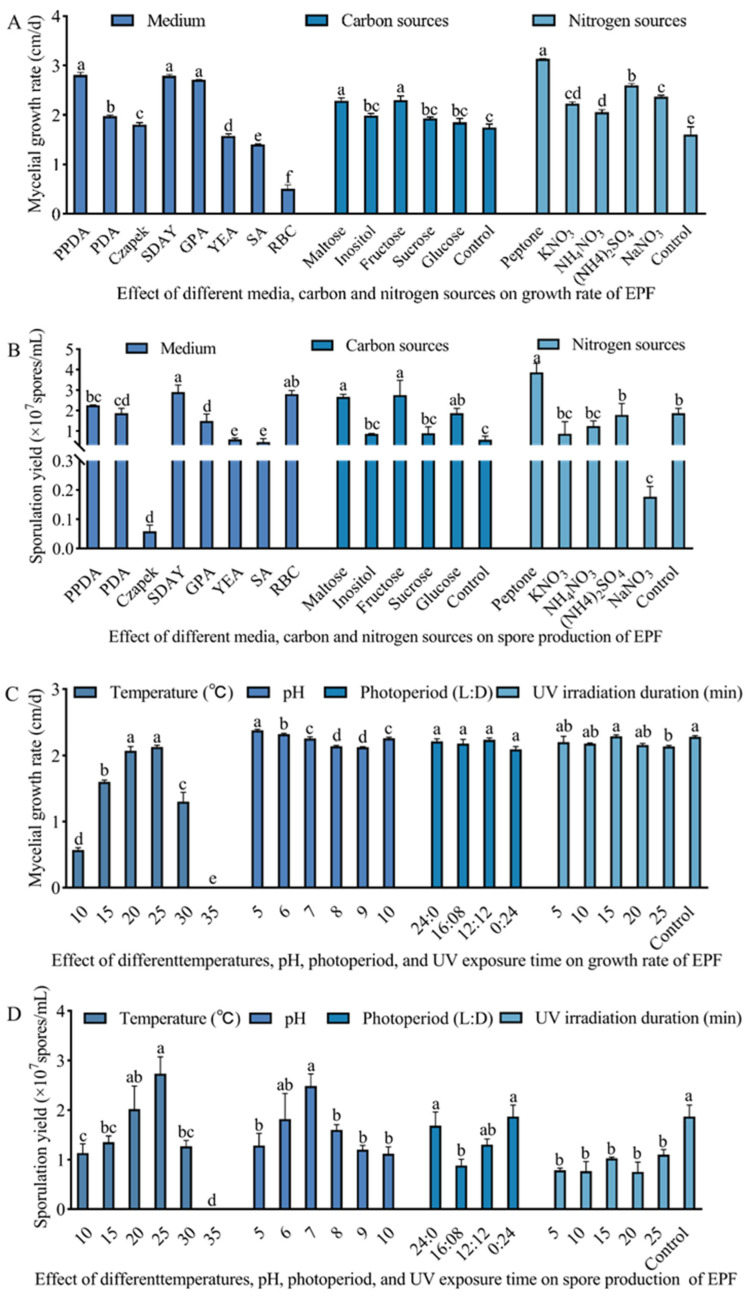
Effect of different media, carbon, and nitrogen sources on (**A**) growth rate and (**B**) spore production of EPF. Effect of different temperatures, pH, photoperiod, and UV exposure time on (**C**) fungal growth rate and (**D**) spore production. Data are mean ± SE. Letters on the error bars indicate significant differences analyzed by ANOVA with the LSD test (*p* < 0.05).

We found marked differences in the growth and sporulation quantities of the *M. hiemalis* on different media (Figure 3A,B). The fastest growth rate of 2.32 cm/d was observed in the Peptone Potato Dextrose Agar (PPDA) medium, followed by the SDAY and Glucose Peptone Agar (GPA) media, which were not significantly different from each other. The slowest mycelial growth was 0.02 cm/d in the Bengal red medium (F_7,16_ = 25.85; *p* < 0.05). The highest sporulation quantity of 2.9 × 10^7^ spores/mL was in the SDAY medium, followed by Bengal red and PPDA media after 6 d of incubation. The lowest spore production of 0.06 × 10^7^ spores/mL was observed in the Czapek–Dox medium, which was significantly less than in other media (F_7,16_ = 358.15; *p* < 0.05). Thus, the SDAY medium was screened as the most suitable medium for the growth and spore production of *M. hiemalis* QH01.

As for the carbon source, strain QH01 grew fastest on medium with fructose, with a growth rate of up to 2.30 cm/d (Figure 3A,B). Mycelial growth was also enhanced with maltose and glucose, with a significant increase compared to the control group (F_5,12_ = 13.23; *p* < 0.05). The fungus produced the most spores on a fructose-containing medium, followed by media containing glucose or maltose, both of which were significantly better than the control (F_5,12_ = 7.73; *p* < 0.05). The growth and sporulation quantity of the fungus on the sucrose- or inositol-supplemented medium were not significantly different from the control medium.

Regarding the nitrogen sources, the fungal mycelial growth rate on the peptone-supplemented medium was 3.13 cm/d, which was significantly higher than for the other nitrogen sources (such as KNO_3_, NaNO_3_, (NH_4_)_2_SO_4_, and NH_4_NO_3_, F_5,12_ = 51.37; *p* < 0.05). The fungus produced the most spores on a peptone-containing medium, while the medium containing KNO_3_, (NH_4_)_2_SO_4_, or NH_4_NO_3_ was not significantly different from the control group. Notably, the spore production was severely inhibited and lower than the control when the fungus was grown with NaNO_3_ as the nitrogen source (F_5,11_ = 11.53; *p* < 0.05). Thus, fructose and peptone were selected as the optimum carbon and nitrogen sources for the growth and spore production of strain QH01 (Figure 3A,B).

Strain QH01 was able to grow from 10 to 30 °C, but the difference was significant (Figure 3C,D). At 25 °C, the fastest growth rate was 2.13 cm/d (F_5,12_ = 156.02; *p* < 0.05). The slowest fungal growth was at 10 °C. Under optimal temperature of 25 °C, the fungus produced 2.73 × 10^7^ spores/mL (F_5,12_ = 12.72; *p* < 0.05). No mycelial growth or spore production was observed at 35 °C.

Medium pH exerted a significant influence on both fungal mycelial growth and sporulation. The maximum mycelial growth rate of 2.38 cm/d was recorded at pH 5.0 (F_5,12_ = 35.18; *p* < 0.05). The optimum pH for fungal spore production was 6–7, but a too-acidic or alkaline medium reduced spore production (F_5,12_ = 3.70; *p* < 0.05). No significant differences in fungal mycelial growth rates were observed across the tested photoperiods (F_3,8_ = 2.02; *p* > 0.05). In contrast, sporulation quantity was affected under different treatments (F_3,8_ = 4.76; *p* < 0.05). The highest spore production (0.87 × 10^7^ spores/mL) was in the dark (24 h dark), and the lowest (0.88 × 10^7^ spores/mL) was at 16L:8D. The mycelial growth rate remained unaffected, showing no significant difference from the control treatment after the fungus was irradiated with UV-C light for 5–20 min (F_5,9_ = 1.91; *p* > 0.05). Compared to the control treatment, however, the sporulation quantity was obviously reduced, and there was little difference between different time treatments (F_5,10_ = 7.47; *p* < 0.05) (Figure 3C,D).

### 3.3. E. extimalis Larvae Infestation with M. hiemalis QH01

**Figure 4:**
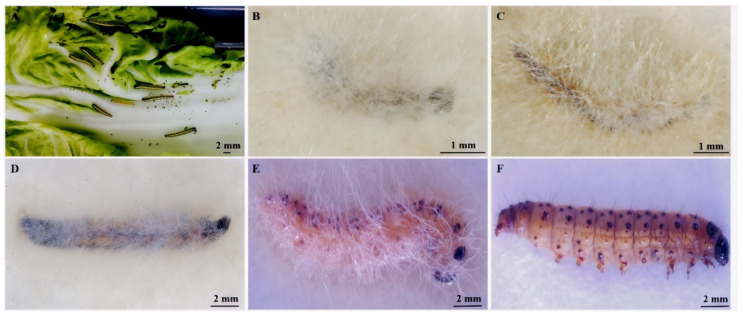
Symptoms of *E. extimalis* larvae infested with *M. hiemalis* QH01. Images were taken after 5 days of moisturized incubation. (**A**) Healthy, (**B**) 1st instar, (**C**) 2nd instar, (**D**) 3rd instar, (**E**) 4th instar, and (**F**) 5th instar larvae.

Since *E. extimalis* larvae were infected by *M. hiemalis* QH01, their feeding rate gradually decreased. The dead larvae turned brown or black-red, and the body softened and finally dried up. The larvae body surface became covered with white mycelium and black sporangia after 2–5 days of moisturized incubation at 25 °C (Figure 4).

### 3.4. Pathogenicity of M. hiemalis QH01 Against E. extimalis Larvae

**Figure 5:**
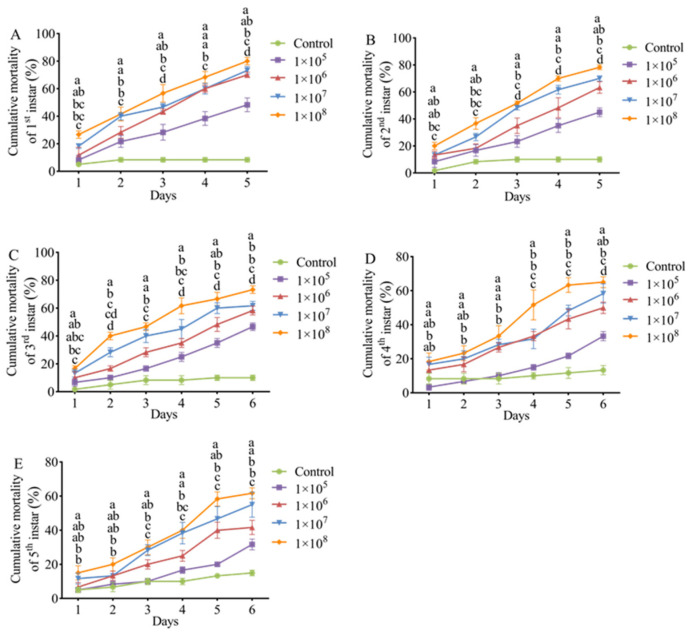
Pathogenicity of *M. hiemalis* QH01 against *E. extimalis* larvae. (**A**–**E**) The mortality rate trend over time (in days) after insect treatment with four different concentrations of *M. hiemalis*. Data are mean ± SE. Letters on the error bars indicate significant differences analyzed using ANOVA with the LSD test (*p* < 0.05).

*E. extimalis* larvae infected with *M. hiemalis* spore suspensions exhibited significant dose- and time-dependent mortality compared to the control group (Figure 5). The spores induced mortality in all ages of insect larvae.

The LC_50_ values of *M. hiemalis* QH01 spores against *E. extimalis* larvae of various ages are listed in Table 1. The LC_50_ exhibited a time-dependent decrease, with larval susceptibility showing an inverse relationship to developmental maturity (first instar > second instar > third instar > fourth instar > fifth instar). Basically, the younger larvae were more sensitive to *M. hiemalis* QH01.

The LT_50_ values of *M. hiemalis* QH01 against *E. extimalis* larvae progressively decreased with increasing inoculum concentration. The fungus exhibited the fastest lethal effect against the 1st–5th instar larvae treated with 1 × 10^8^ spores/mL suspensions, and LT_50_ values were 2.99, 3.01, 3.37, 4.04, and 4.40 days, respectively. In contrast, under 1 × 10^5^ spores/mL concentration, the fungus had the slowest lethal effect, and LT_50_ values were 5.15, 5.38, and 5.29 days for 1st–3rd instar larvae. With this spore concentration, the mortality rate of 4th-5th instar larvae was lower than 50% even after 6 days of exposure, and it was not possible to estimate the LT_50_ (Table 2). These results indicated that *M. hiemalis* QH01 is highly virulent to young larvae.

### 3.5. Greenhouse Experiments

**Figure 6:**
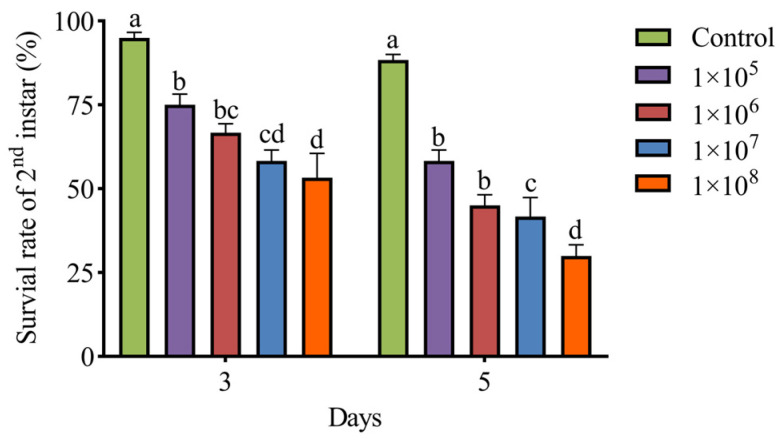
Greenhouse pot tests were performed to determine the survival rate of second instar larvae of *E. extimalis* against *M. hiemalis*. Data are mean ± SE. Letters on the error bars indicate significant differences analyzed using ANOVA with the LSD test (*p* < 0.05).

The results of greenhouse pot experiments of *M. hiemalis* QH01 on second instar larvae of *E. extimalis* are shown in Figure 6. By spraying *M. hiemalis* suspensions of 1 × 10^5^, 1 × 10^6^, 1 × 10^7^, and 1 × 10^8^ spores/mL, the survival rates of second instar larvae at 5 days were 58.33%, 45%, 41.67%, and 30%, respectively, which were significantly lower than control group (F_4,15_ = 37.47; *p* < 0.05). The suspension containing 1 × 10^8^ spores/mL resulted in the lowest survival rate, indicating a concentration-dependent survival.

## 4. Discussion

The composition of the culture medium can affect the growth characteristics of EPF [25]. We found that the SDAY medium was the most suitable for the growth and spore production of *M. hiemalis* QH01, which was consistent with previous studies [26]. The spore yield of other tested media, such as PDA, GPA, Yeast extract agar (YEA), etc., was relatively lower than that of SDAY, which might have been due to the difference in selective absorption of nutrients by the same strain because of the different types, contents, and solubility states of nutrients in different media. In addition, carbon and nitrogen sources can also affect the growth and spore production of EPF [27]. This study demonstrated that fructose favored the growth and spore production of *M. hiemalis* QH01, while the absence of a carbon source inhibited it. The best growth and spore production of strain QH01 were observed with peptone as a nitrogen source, which was consistent with Huang et al. [28].

The optimal growth and reproduction of EPF cannot be achieved without appropriate environmental conditions [29]. Temperature affects the metabolic activity of entomopathogenic fungi through key physiological processes including enzyme biosynthesis, toxin production, spore germination, and germ tube penetration [30]. We found that *M. hiemalis* QH01 exhibited a growth and sporulation temperature range of 10–30 °C, with an optimum at 25 °C. In contrast, *M. hiemalis* BO-1 grew the fastest at 23 °C and produced the highest sporulation at 18 °C [31]. This is likely attributable to QH01’s specific adaptations to the extreme climatic conditions of its native Tibetan Plateau environment. The medium pH can significantly impact the growth and pathogenicity of EPF [32]. For example, pH 5.5–6.5 is best suited for the sporulation of *B. bassiana*, and the same is also good for its pathogenicity [33,34]. *M. hiemalis* QH01 can grow and produce spores in a wide range of pH (5–10), which is consistent with the results of Wang et al. [35]. In addition to temperature and pH, external conditions such as natural light have a certain impact on EPF. In this study, *M. hiemalis* had the highest sporulation in total darkness. This may be attributed to *Mucor* spp.’s natural adaptations to light-limited environments, given their predominant colonization of soil and decaying organic substrates [16]. Their spore production mechanism may have evolved a dependence on darkness. UV light usually inhibits the growth, sporulation, and pathogenicity of EPF. We found that UV irradiation decreased spore production in *M. hiemalis*. Yao et al. [36] reported that 312 and 365 nm UV light exposure decreased the pathogenicity of *M. anisopliae* against tobacco aphids. The influence of ultraviolet light on the pathogenicity of *M. hiemalis* had to be considered when applying it to pest control in the field.

So far, there have been few reports on the biological control of *E. extimalis*. Wang et al. [37] conducted a field investigation and reported that the Cloth beetle and *B. bassiana* were the natural enemies of the insect in the larval stage, while the pupal stage could be parasitized by mites. Here, we show the pathogenicity of *M. hiemalis* against *E. extimalis*. Particularly, the young larvae of *E. extimalis* were more susceptible to *M. hiemalis* QH01, while the older larvae exhibited relatively a lower mortality rate. This difference can be attributed to the different body wall structures and immune capacities of young and old larvae. A thinner body wall in younger larvae makes them more susceptible to EPF infection. Older larvae are more resistant to disease due to hardening and thickening of the cuticle, enhanced immunity and metabolism [38]. Moreover, with the increase in the developmental stage of *E. extimalis*, the corresponding LT_50_ and LC_50_ values increased for the same concentration of *M. hiemalis*. This indicated that *M. hiemalis* QH01 is more virulent to lower-stage larvae than the developed larvae. This result is in agreement with the findings of Tozlu et al. [39]. Zhu et al. [40] reported 46.25% mortality in *Bradysia impatiens* second instar larvae following 5 days of exposure to *M. hiemalis* BO-1 at 1 × 10^6^ spores/mL, whereas our experimental data demonstrated 63.33% mortality in *E. extimalis* larvae under identical spore concentration with *M. hiemalis* QH01. This may be the result of host specificity, environmental adaptation, etc., which led to significant differences in the pathogenicity of strains of the same species. Notably, surviving larvae exhibited significantly reduced feeding activity, progressed to the prepupal stage with sporadic pupation, but ultimately failed to complete adult eclosion. It is possible that infection by *M. hiemalis* QH01 induces a sublethal effect on life history traits including developmental processes and reproduction in *E. extimalis* [41]. EPF can target all developmental stages of insects, such as eggs, larvae, and adults. Wang et al. [42] isolated a strain of *M. hiemalis* from diseased *Bradysia odoriphaga* larvae and found its pathogenicity varied against different stages of insect; strong pathogenicity was against larvae and relatively weak pathogenicity was against eggs and pupae. In the study, the pathogenicity of *M. hiemalis* QH01 was determined only against the larvae of *E. extimalis*, and further studies are needed to determine the pathogenicity against other insect forms.

Most EPF performs remarkably well under controlled conditions, such as in the laboratory, exhibiting high mortality against the target insects. However, abiotic factors such as changes in temperature, humidity, and UV exposure can significantly affect the biocontrol effect of EPF in field applications [43]. In greenhouse environments, after 7 days of treatment with 2 × 10^6^ spores/mL of Bea111 (*B. bassiana*) and 9 × 10^6^ spores/mL of Isa340 (*Isaria javanica*), third instar larvae of *Duponchelia fovealis* exhibited 52% and 45% mortality rates, respectively [44]. The larval stage of *E. extimalis* causes the most damage and economic loss in rapeseed fields. First instar larvae exhibit higher mortality rates when attempting to bore into rapeseed pods for feeding. From the third instar onward, larvae exhibit migratory behavior, transitioning from pod-boring to stem-tunneling and inter-plant movement, while their food consumption surges dramatically [45]. Thus, we evaluated the biocontrol potential of *M. hiemalis* QH01 against the second instar larvae of *E. extimalis* under greenhouse conditions and found it significantly reduced their survival rates.

In this study, the effectiveness of strain QH01 against *E. extimalis* second instar larvae in greenhouse pots differed from the results of the indoor virulence assay, which may be due to different bioassay methods [46] and external environmental conditions. To sum up, when preparing fungal powder, ultraviolet protectants, thermal stabilizers, and ionic dispersants should be added to the spore powder to enhance environmental adaptability. Greenhouse and field applications should be carried out after sunset or on cloudy days when the temperature is around 20–25 °C.

## 5. Conclusions

In the present study, a fungal strain isolated from *E. extimalis* larvae was identified as *M. hiemalis* QH01 based on morphological characteristics, ITS, and 18S rDNA sequence analyses. To our knowledge, this is the first report assessing the biocontrol effects of an entomopathogenic fungus against *E. extimalis*. Our results lay the foundation for the subsequent research on designing a green prevention and control program for *E. extimalis* using EPF to benefit the spring rapeseed industry.

## Figures and Tables

**Table 1:** Virulence regression equations for LC_50_ values of *M. hiemalis* QH01 against *E. extimalis* larvae.

Days	Developmental Stage	Virulence Regression Equation	LC_50_ (Spores/mL)	95% CI (Spores/mL)
3	1st	Y = 0.229X − 1.650	1.63 × 10^7^	3.21 × 10^6^–6.85 × 10^8^
	2nd	Y = 0.262X − 1.982	3.57 × 10^7^	8.00 × 10^6^–1.30 × 10^9^
	3rd	Y = 0.253X − 1.917	3.88 × 10^7^	8.16 × 10^6^–2.11 × 10^9^
	4th	Y = 0.238X − 2.259	3.13 × 10^9^	1.63 × 10^8^–3.89 × 10^15^
	5th	Y = 0.241X − 2.362	6.53 × 10^9^	2.59 × 10^8^–5.93 × 10^16^
4	1st	Y = 0.233X − 1.340	5.71 × 10^5^	1.14 × 10^4^–2.82 × 10^6^
	2nd	Y = 0.312X − 1.918	1.40 × 10^6^	2.69 × 10^5^–4.73 × 10^6^
	3rd	Y = 0.275X − 1.720	1.83 × 10^6^	2.89 × 10^5^–7.72 × 10^6^
	4th	Y = 0.312X − 2.499	1.01 × 10^8^	2.27 × 10^7^–3.40 × 10^9^
	5th	Y = 0.288X − 2.387	1.93 × 10^8^	3.38 × 10^7^–2.33 × 10^10^
5	1st	Y = 0.289X − 1.412	7.77 × 10^4^	1.07 × 10^3^–4.00 × 10^5^
	2nd	Y = 0.294X − 1.528	1.58 × 10^5^	5.15 × 10^3^–6.83 × 10^5^
	3rd	Y = 0.267X − 1.525	5.23 × 10^5^	2.92 × 10^4^–2.14 × 10^6^
	4th	Y = 0.344X − 2.397	9.13 × 10^6^	3.05 × 10^6^–4.22 × 10^7^
	5th	Y = 0.326X − 2.355	1.69 × 10^7^	5.24 × 10^6^–1.21 × 10^8^

**Table 2:** Virulence regression equations for LT_50_ values of *M. hiemalis* QH01 against *E. extimalis* larvae.

Concentration (Spores/mL)	Developmental Stage	Virulence Regression Equation	LT_50_ (Days)	95% CI (Days)
1 × 10^5^	1st	Y = 0.265X − 1.365	5.15	4.38–12.86
	2nd	Y = 0.300X − 1.612	5.38	4.61–10.39
	3rd	Y = 0.296X − 1.564	5.29	4.54–10.20
	4th	#	#	#
	5th	#	#	#
1 × 10^6^	1st	Y = 0.347X − 1.186	3.41	2.00–3.95
	2nd	Y = 0.363X − 1.481	4.08	3.46–4.80
	3rd	Y = 0.298X − 1.262	4.24	3.51–5.63
	4th	#	#	#
	5th	#	#	#
1 × 10^7^	1st	Y = 0.353X − 1.148	3.25	1.63–3.80
	2nd	Y = 0.299X − 1.068	3.57	1.71–4.21
	3rd	Y = 0.275X − 1.072	3.90	2.46–4.90
	4th	#	#	#
	5th	#	#	#
1 × 10^8^	1st	Y = 0.421X − 1.261	2.99	1.60–3.51
	2nd	Y = 0.391X − 1.176	3.01	1.39–3.55
	3rd	Y = 0.373X − 1.258	3.37	2.12–3.88
	4th	Y = 0.385X − 1.557	4.04	3.45–4.66
	5th	Y = 0.367X − 1.614	4.40	3.88–5.40

Note: # indicates that the mortality rate of *E. extimalis* larvae was below 50%, and LT_50_ values cannot be estimated.

## Data Availability

Data are contained within the article.

## Supplementary Materials

The following supporting information can be downloaded at: https://www.mdpi.com/article/10.3390/biology14050467/s1. Table S1: Test medium composition.

## References

[1] Shao H.N., Liu Y.X., Liu Y.J., Lai Y.P. (2022). The Effect of Ice-Nucleation-Active Bacteria on Metabolic Regulation in *Evergestis extimalis* (Scopoli) (Lepidoptera: Pyralidae) Overwintering Larvae on the Qinghai-Tibet Plateau. Insects.

[2] Lai Y.P., Tao K., Hou T.P. (2019). Preliminary analysis of geographical distribution based on cold hardiness for *Evergestis extimalis* (Scopoli) (Lepidoptera: Pyralidae) on Qinghai-Tibet Plateau. Entomol. Res..

[3] Shah P.A., Pell J.K. (2003). Entomopathogenic fungi as biological control agents. Appl. Microbiol. Biotechnol..

[4] Samuels R.I., Coracini D., Martins dos Santos C.A., Gava C.A.T. (2002). Infection of *Blissus antillus* (Hemiptera: Lygaeidae) eggs by the entomopathogenic fungi *Metarhizium anisopliae* and *Beauveria bassiana*. Biol. Control.

[5] Yao H.Q., Xie Y.P., Wang E.H. (2015). Morphologic observation of *Apocheima cinerarius Erschoff* (Lepidoptera: Geometridae) pupae and comparison of virulence of *Beauveria bassiana* infected on them. J. Environ. Entomol..

[6] Cruz-Avalos A.M., Bivián-Hernández M.L.Á., Ibarra J.E., Del Rincón-Castro M. (2019). High virulence of mexican entomopathogenic fungi against fall armyworm, (Lepidoptera: Noctuidae). J. Econ. Entomol..

[7] Shehzad M., Tariq M., Mukhtar T., Gulzar A. (2021). On the virulence of the entomopathogenic fungi, *Beauveria bassiana* and *Metarhizium anisopliae* (Ascomycota: Hypocreales), against the diamondback moth, *Plutella xylostella* (L.) (Lepidoptera: Plutellidae). Egypt. J. Biol. Pest Control.

[8] Xie N., Yin Y.P., Zhang J.W., Shen J.F., Wang Z.K. Characterization of the *Metarhizium anisopliae* CQMa128 and its virulence measurements against Lepidoptera insects. Proceedings of the 2009 Annual Meeting of the Mycological Society of China.

[9] Varela A., Morales E. (1996). Characterization of some *Beauveria bassiana* isolates and their virulence toward the Coffee Berry Borer, *Hypothenemus hampei*. J. Invertebr. Pathol..

[10] Wraight S.P., Inglis G.D., Goettel M.S. (2007). Fungi. Field Manual of Techniques in Invertebrate Pathology: Application and Evaluation of Pathogens for Control of Insects and other Invertebrate Pests.

[11] Steinkraus D.C. (2006). Factors affecting transmission of fungal pathogens of aphids. J. Invertebr. Pathol..

[12] Alali S., Mereghetti V., Faoro F., Bocchi S., Al Azmeh F., Montagna M. (2019). Thermotolerant isolates of *Beauveria bassiana* as potential control agent of insect pest in subtropical climates. PLoS ONE.

[13] Jaronski S.T. (2010). Ecological factors in the inundative use of fungal entomopathogens. BioControl.

[14] Mann A.J., Davis T.S. (2020). Plant secondary metabolites and low temperature are the major limiting factors for *Beauveria bassiana* (Bals.–Criv.) Vuill. (Ascomycota: Hypocreales) growth and virulence in a bark beetle system. BioControl.

[15] Lyu M.L., Liu Z., Song Z., Wang Y.N., Liu X.Y. (2019). Diversity and distribution of culturable Mucoromycota fungi in the Greater Khinggan Mountains. J. Biodivers. Sci..

[16] Morrissey C.O., Kim H.Y., Garnham K., Dao A., Chakrabarti A., Perfect J.R., Alastruey-Izquierdo A., Harrison T.S., Bongomin F., Galas M. (2024). Mucorales: A systematic review to inform the World Health Organization priority list of fungal pathogens. Med. Mycol..

[17] Nentwig W., Prillinger H. (1990). A zygomycetous fungus as a mortality factor in a laboratory stock of spiders. J. Arachnol..

[18] Heitor F. (1962). Wound parasitism by the fungus *Mucor hiemalis* Wehmer in insects. Ann. Epiphyt..

[19] Reiss J. (1993). Biotoxic activity in the Mucorales. Mycopathologia.

[20] Konstantopoulou M.A., Mazomenos B.E. (2005). Evaluation of *Beauveria bassiana* and *B. brongniartii* strains and four wildtype fungal species against adults of *Bactrocera oleae* and *Ceratitis capitata*. BioControl.

[21] Konstantopoulou M.A., Milonas P., Mazomenos B.E. (2006). Partial purification and insecticidal activity of toxic metabolites secreted by a *Mucor hiemalis* strain (SMU-21) against adults of *Bactrocera oleae* and *Ceratitis capitata* (Diptera: Tephritidae). J. Econ. Entomol..

[22] Hallouti A., Ait Hamza M., Zahidi A., Ait Hammou R., Bouharroud R., Ait Ben Aoumar A., Boubaker H. (2020). Diversity of entomopathogenic fungi associated with Mediterranean fruit fly (*Ceratitis capitata* (Diptera: Tephritidae)) in Moroccan Argan forests and nearby area: Impact of soil factors on their distribution. BMC Ecol..

[23] Wei J.C. (1979). Fungi Identification Manual.

[24] Kumar S., Nei M., Dudley J., Tamura K. (2008). MEGA: A biologist-centric software for evolutionary analysis of DNA and protein sequences. Brief. Bioinform..

[25] Fargues J.F., Robert P.H. (1983). Effect of passaging through scarabeid hosts on the virulence and host specificity of two strains of the entomopathogenic hyphomycete *Metarhizium anisopliae*. Can. J. Microbiol..

[26] Banu J.G., Rajalakshmi S. (2014). Standardisation of media for mass multiplication of entomopathogenic fungi. Indian J. Plant Prot..

[27] Moore D., Douro-Kpindou O.K., Jenkins N.E., Lomer C.J. (1996). Effects of moisture content and temperature on storage of *Metarhizium flavoviride* conidia. Biocontrol Sci. Technol..

[28] Huang P., Yao J.A., Yu D.Y. (2018). Biological Characteristics of *Metarhizium anisopliae* FM−03 and Its Infection against *Planococcus citri*. Chin. J. Biol. Control.

[29] Hou Y., Xia Y.F., Xu J.Q. (2015). Identification and Biological Characteristics of a *Metarhizium* Strain and Its Virulence against Oriental Migratory Locust. Chin. J. Biol. Control.

[30] Anwar A.U., Xia S., Meng L., Fahim R.M., Zhang Z.Y., Zhang H.Y. (2021). Isolation, characterization, culturing, and formulation of a new *Beauveria bassiana* fungus against *Diaphorina citri*. BioControl.

[31] Zhu G.D., Ding W.J., Xue M., Li Z., Li M., Zhao Y. (2022). Identification and Pathogenicity of a New Entomopathogenic Fungus, *Mucor hiemalis* (Mucorales: Mucorales), on the Root Maggot, *Bradysia odoriphaga* (Diptera: Sciaridae). J. Insect Sci..

[32] St leger R., Nelson J.O., Screen S.E. (1999). The entomopathogenic fungus *Metarhizium anisopliae* alters ambient pH, allowing extracellular protease production and activity. Microbiology.

[33] Mishra S., Kumar P., Malik A. (2015). Effect of temperature and humidity on pathogenicity of native *Beauveria bassiana* isolate against *Musca domestica* L.. J. Parasit. Dis..

[34] Cheng G.H., Shu J., Ding K.J. (2006). Study on Nutrition Demand and Culture Condition of *Beauveria bassiana*. Chin. Agric. Sci. Bull..

[35] Wang B., Fan M.Z., Li Z.Z. (2000). Sieving of Selective Media for *Beauveria bassiana*. J. Anhui Agric. Univ..

[36] Yao S.L., Ying S.H., Feng M.G., Hatting J.L. (2010). In vitro and in vivo responses of fungal biocontrol agents to gradient doses of UV-B and UV-A irradiation. BioControl.

[37] Wang L.Y., Sun Q., Lin Z.W., Wang H.Z., Wu Q.S., Xin H.P., Zhong X.Z. (1998). Occurrence and control of *Evergestis extimalis*. Chin. Bull. Entomol..

[38] Strak J.D., Sherman M. (1989). Toxicity, penetration, and metabolism of acephate in three fruit fly species (Diptera: Tephritidae). J. Econ. Entomol..

[39] Tozlu E., Tozlu G., Kotan R. (2022). Biocontrol of *Cymbalophora rivularis* (Menetries) (Lepidoptera: Erebidae) larvae by entomopathogenic bacteria and fungi. Egypt. J. Biol. Pest Control.

[40] Zhu G.D., Ding W.J., QIU J.Y., Wang Y., Xue M., Zhao H.P., Zhang G.F. (2024). Pathogenicity and field control efficiency of *Mucor hiemalis* BO-1 against *Bradysia odoriphaga* Larvae. Chin. J. Biol. Control..

[41] Latifian M., Soleimannejadian E., Ghazavi M., Mosadegh M.S., Hayati J. (2010). Effects of sublethal concentrations of fungus *Beauveria bassiana* on the reproductive potentials of sawtoothed beetle *Oryzaephilus surinamensis* on commercial date cultivars. Plant Prot. J..

[42] Wang Y. (2021). Poisoning Activity of a New Strain of *Mucor hiemalis* to *Bradysia odoriphaga* Yang et Zhang. Master’s Thesis.

[43] Beris E., Papachristos D., Ponchon M., Caca D., Kontodimas D., Reineke A. (2024). The effects of temperature on pathogenicity of entomopathogenic fungi for controlling larval populations of the European grapevine moth (*Lobesia botrana*) (Lepidoptera: Tortricidae). J. Crop Prot..

[44] Amatuzzi R.F., Poitevin C.G., Poltronieri A.S., Zawadneak M.A.C., Pimentel I.C. (2018). Susceptibility of *Duponchelia fovealis* Zeller (Lepidoptera: Crambidae) to Soil-Borne Entomopathogenic Fungi. Insects.

[45] Xie C.H. (2019). Study on Occurrence and Control Countermeasures of Harmful Organisms with Rapeseed in Huangzhong County of Qinghai. Master’s Thesis.

[46] Liu H., Skinner M., Parker B.L. (2003). Bioassay method for assessing the virulence of *Beauveria bassiana* against tarnished plant bug, *Lygus lineolaris* (Hem., Miridae). J. J Appl Entomol..

