# Supplementary material for: Isolation, Identification, Biological Characterization, and Pathogenicity of Entomopathogenic Fungus from the Larvae of the Evergestis extimalis (Scopoli) (Lepidoptera: Pyralidae)
Source: Biology (Basel). 2025 Apr 25;14(5):467. doi: 10.3390/biology14050467 (PMC12109197; doi:10.3390/biology14050467)
Supplement: Supplementary file 1 [file biology-14-00467-s001.zip › biology-3577144-supplementary.pdf]

Table S1 Test medium composition

Medium	Components
Starch agar (SA)	Soluble starch 40 g·L^{-1}, yeast extract 5 g·L^{-1}, and agar 20 g·L^{-1}
Czapek-Dox	Fructose 30.00 g·L^{-1}, K_2HPO_4 1.00 g·L^{-1}, $MgSO_4$ 0.50 g·L^{-1}, KCl 0.50 g·L^{-1}, $NaNO_3$ 3.00 g·L^{-1}, $FeSO_4·7H_2O$ 0.01 g·L^{-1}, and agar 20.00 g·L^{-1}
Glucose peptone agar (GPA)	Peptone 10 g·L^{-1}, dextrose 40 g·L^{-1}, and agar 20 g·L^{-1}
Potato dextrose agar (PDA)	Peeled potato 200 g·L^{-1}, dextrose 20 g·L^{-1}, and agar 18 g·L^{-1}
Rose Bengal medium (RBC)	Peptone 5.0 g·L^{-1}, dextrose 10.0 g·L^{-1}, KH_2PO_4 1.0 g·L^{-1}, MSO_4 0.5 g·L^{-1}, 1/3000 Bengal red 100 mL·L^{-1}, and agar 18.0 g·L^{-1}
Sabouraud dextrose agar + yeast (SDAY)	Dextrose 40 g·L^{-1}, yeast extract 10 g·L^{-1}, peptone 10 g·L^{-1}, and agar 20 g·L^{-1}
Peptone potato dextrose agar (PPDA)	Peeled potato 200 g·L^{-1}, dextrose 20 g·L^{-1}, peptone 20 g·L^{-1}, and agar 18 g·L^{-1}
Yeast extract agar (YEA)	Dextrose 4 g·L^{-1}, yeast extract 4 g·L^{-1}, maltose 10 g·L^{-1}, and agar 20 g·L^{-1}
